# Supplementary material for: Pupil size modulation drives retinal activity in mice and shapes human perception
Source: Nat Commun. 2025 Aug 8;16:7334. doi: 10.1038/s41467-025-62736-4 (PMC12334691; doi:10.1038/s41467-025-62736-4)
Supplement: Supplementary file 1 — Supplementary Information [file 41467_2025_62736_MOESM1_ESM.pdf]

Online content for

Pupil size modulation drives retinal activity in mice and shapes human perception

Tjasa Lapanja[1,2]†, Pietro Micheli[1,3]†, Andrés González-Guerra[1], Oleksandr Radomskyi[1], Gioia De Franceschi[1,4], Anna Muraveva[1], Alexander Attinger[5], Chiara Nina Roth[5], Matteo Tripodi[1], Tom Boissonnet[1], Marina Sabbadini[1,2,6], Josephine Jüttner[7], Petri Ala-Laurila[8,9], Georg Keller[5,10], Gabriel Peinado Allina[8], Hiroki Asari[1], Santiago B. Rompani[1]*

Corresponding author: santiago.rompani@embl.it

file includes:

Figs. S1 to S18

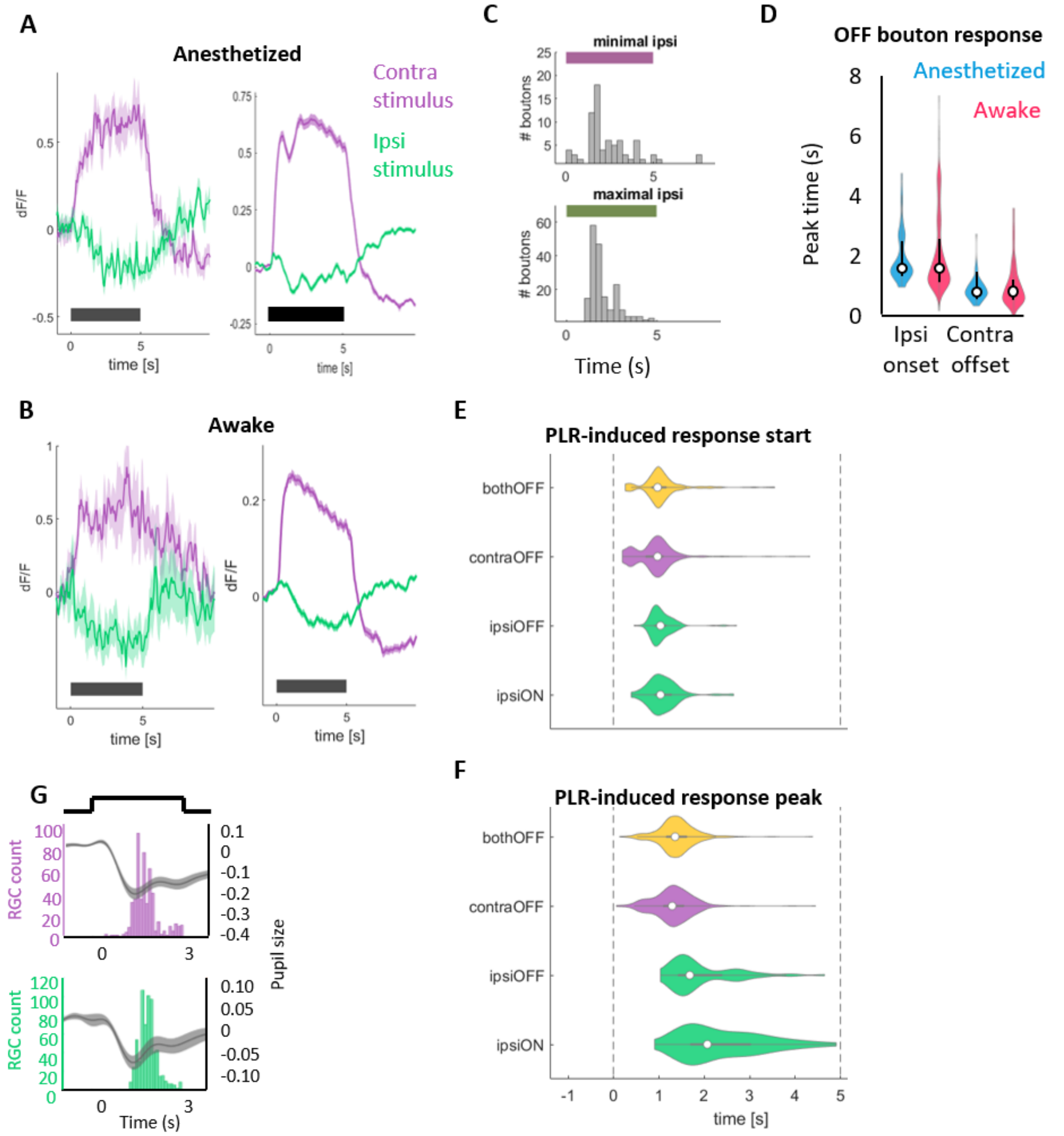

Figure S1: Binocular responses in early visual pathway.
A) Left: example contralateral ON RGC bouton responding to full field flash stimulation to contralateral (violet) or ipsilateral eye (green) Right: ON population response (n=533 boutons, N=5 animals). B) Same as A, but in awake animals (n=693 boutons, N=5 animals). C) Left: Time of minimal response to ipsilateral stimulation in ipsi-responding ON boutons in anesthetized mice (n=101 boutons, N=5 animals). Right: Time to maximal response to ipsilateral stimulation in ipsi-responding OFF boutons (n=192 boutons, N=5 animals). D) Maximal peak latency of OFF boutons responding ipsilateral stimulation (peak during onset) and contralateral stimulation (peak during offset) in awake and anesthetized mice. E) Beginning of PLR-driven response (in seconds after onset) for OFF RGC boutons responding to binocular (yellow, n=314 boutons), contralateral (purple, n=321 boutons) and ipsilateral (green, n=148 boutons) stimulation and ON RGC boutons responding to ipsilateral stimulation (green, n=59 boutons). F) Same as E) for the peak of PLR-driven response. G) Contralateral pupil size change overlaid over peak time of onset response of OFF boutons during contralateral (left) and ipsilateral (right) stimulation.

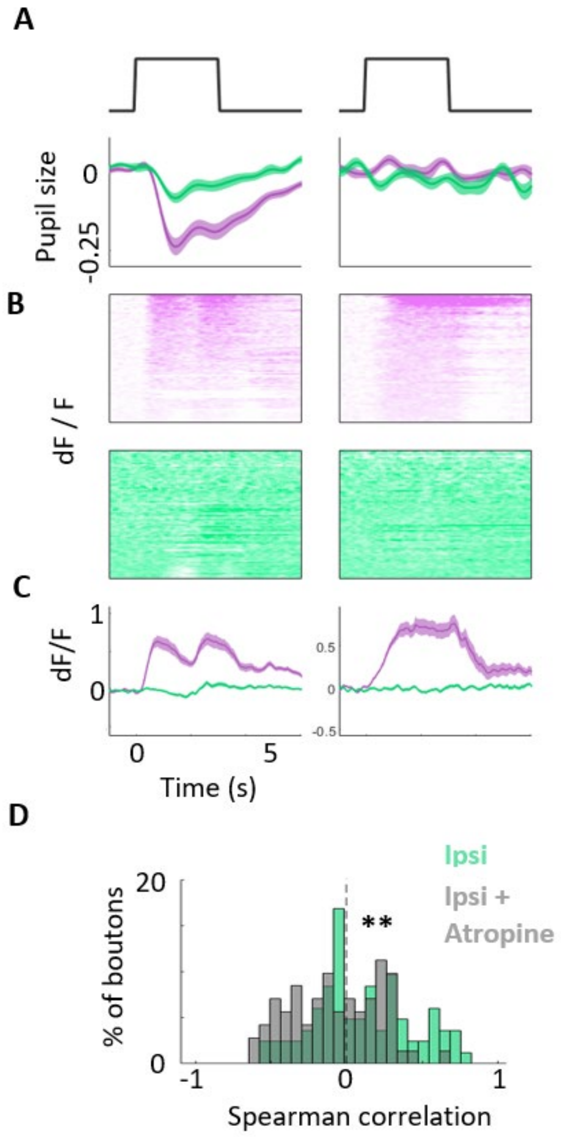

Figure S2: PLR modulates responses of ON RGC boutons. A) Left: Change in pupil size of the right eye due to direct PLR (purple) and consensual PLR (green). Right: Same as left, but after application of the atropine. B) Top Left: Heatmap showing all contralateral ON RGC bouton (n=83 boutons, N=5 animals) responses to contralateral full-field stimulation (scale -0.1 dF/F to 2 dF/F). Bottom left: Same boutons as in B Top left responding to full field flash to the ipsilateral eye (scale -0.5 dF/F to 0.5 dF/F). C) Same as B after the application of atropine to contralateral eye (n=71 boutons, N=5 animals). C) Left: Population response (mean +/- SEM) of boutons presented in B. Response to contralateral (purple) and ipsilateral (green) stimulation. Right: Same as B left, after application of atropine to the contralateral eye. D) Spearman correlation of response to ipsilateral stimulation and pupil constriction before atropine (green) and after atropine (gray). Two-sample t test p=6.13*10^-5.

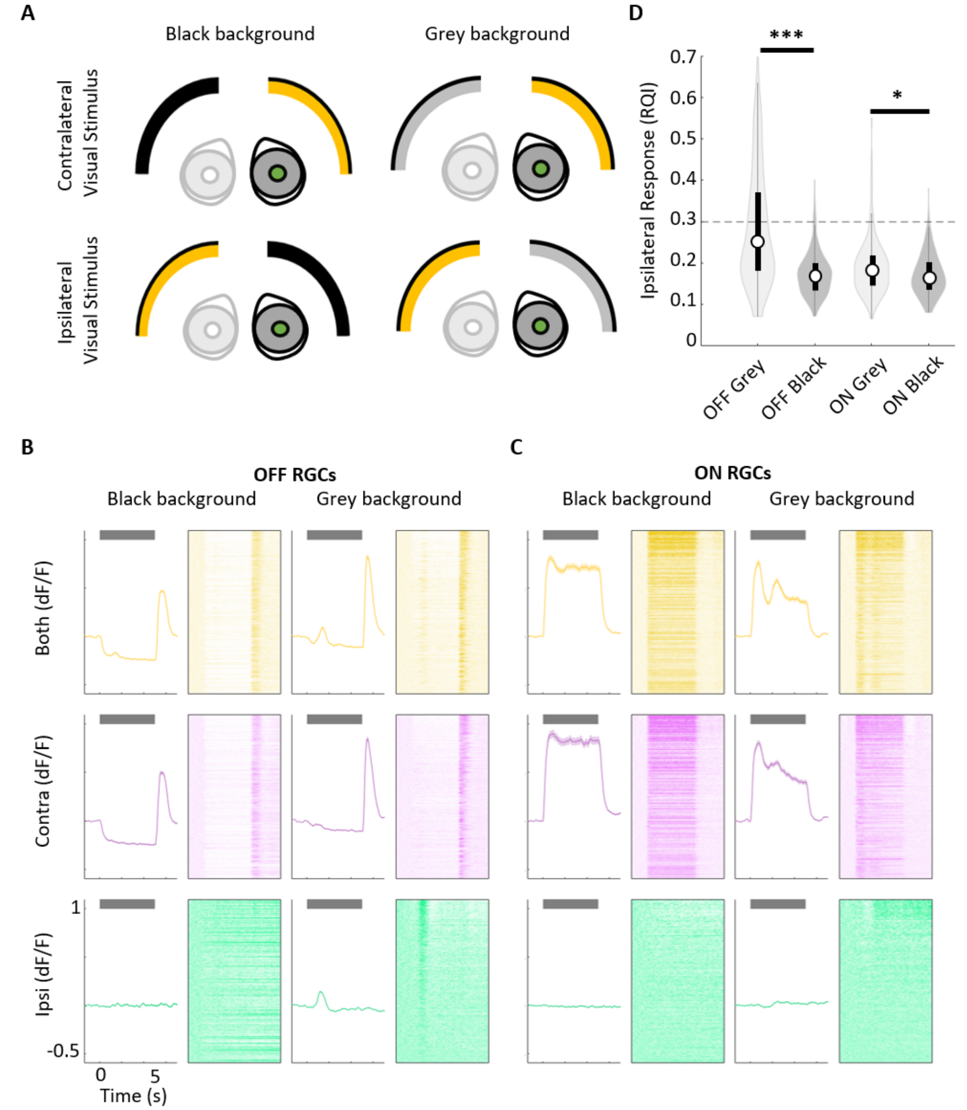

Figure S3: The PLR-driven response depends on background luminance. A) Schematics of the visual stimulation. Left: No background illumination. Either the contralateral or the ipsilateral eye was stimulated with UV LED (300mA). The non-stimulated eye was in the dark. Right: Background illumination. Either the contralateral or the ipsilateral eye was stimulated with UV LED (300mA). The non-stimulated eye was presented with constant background stimulation with UV LED (50mA). B) Population responses and all responses (heatmap) of OFF boutons (n=353 boutons, N=3 animals) to contralateral (violet, top), heatmap range = -0.3dF/F to 2dF/F and ipsilateral (green, bottom), heatmap range = -0.3dF/F to 0.6 dF/F stimulation in no background illumination (left) and background illumination (right) conditions. C) Same as B for ON boutons. n=219 boutons, N=3 animals. D) Response quality index (RQI) for all responses to ipsilateral stimulation. OFF responses background vs. no background p<0.001, OFF responses background vs. no background p=0.002, ANOVA.

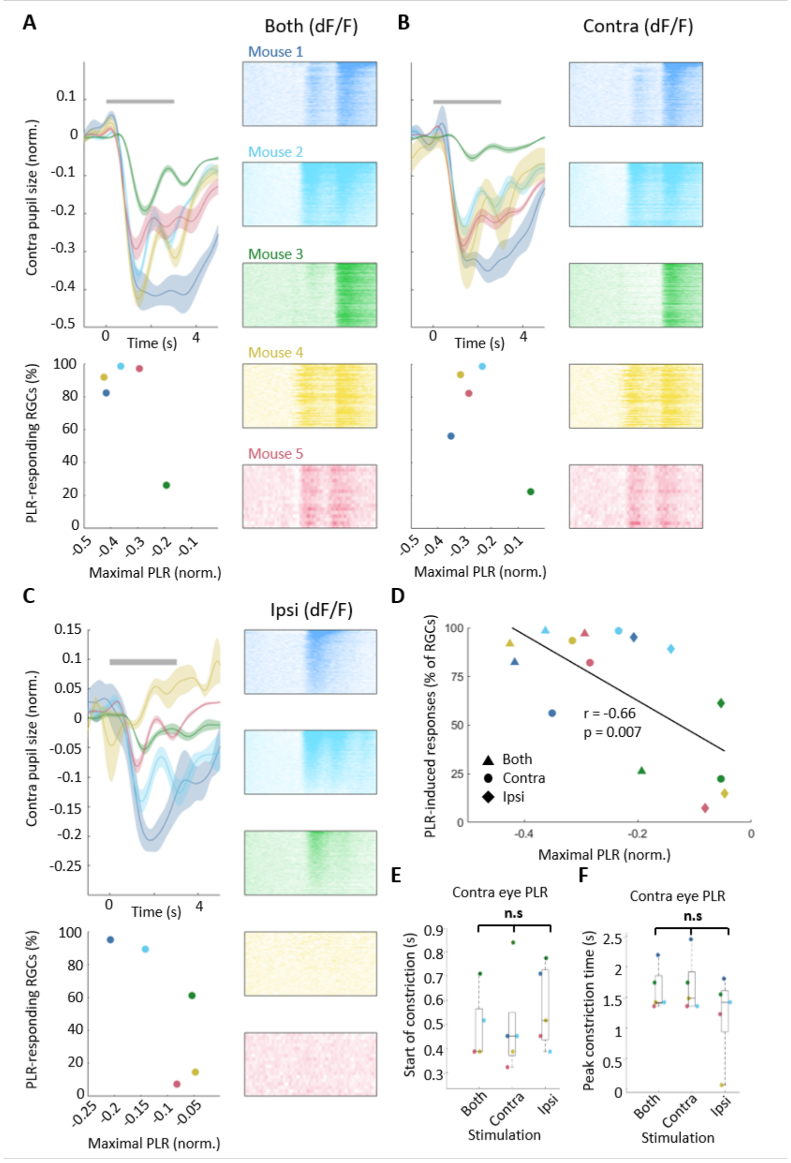

Figure S4: Variability in the strength of PLR and PLR-driven responses. A) Top left: Contralateral pupil size of 5 mice during binocular stimulation. Right: Visual responses of all OFF RGC boutons in each mouse (same color code as in top left). Bottom left: Correlation between maximal PLR (minimal pupil size) and percentage of PLR-driven responses in each mouse. B) Same as A, responses to contralateral stimulation. C) Same as A, responses to ipsilateral stimulation. D) Relationship between maximal PLR and percentage of PLR-driven responses in all animals (color) and all conditions (shape). E) Left: Start of PLR in the contralateral eye (in seconds after start of stimulation) for binocular, contralateral, and ipsilateral stimulation (mean: both=0.48s, contra=0.49s, ipsi=0.57s, ANOVA: $p_{both:contra}=0.99$, $p_{both:ipsi}=0.69$, $p_{ipsi:contra}=0.76$). F) Same as E, but peak PLR time. Mean: both=1.63, contra=1.68, ipsi=1.21 (ANOVA: $p_{both:contra}=0.99$, $p_{both:ipsi}=0.44$, $p_{ipsi:contra}=0.36$).

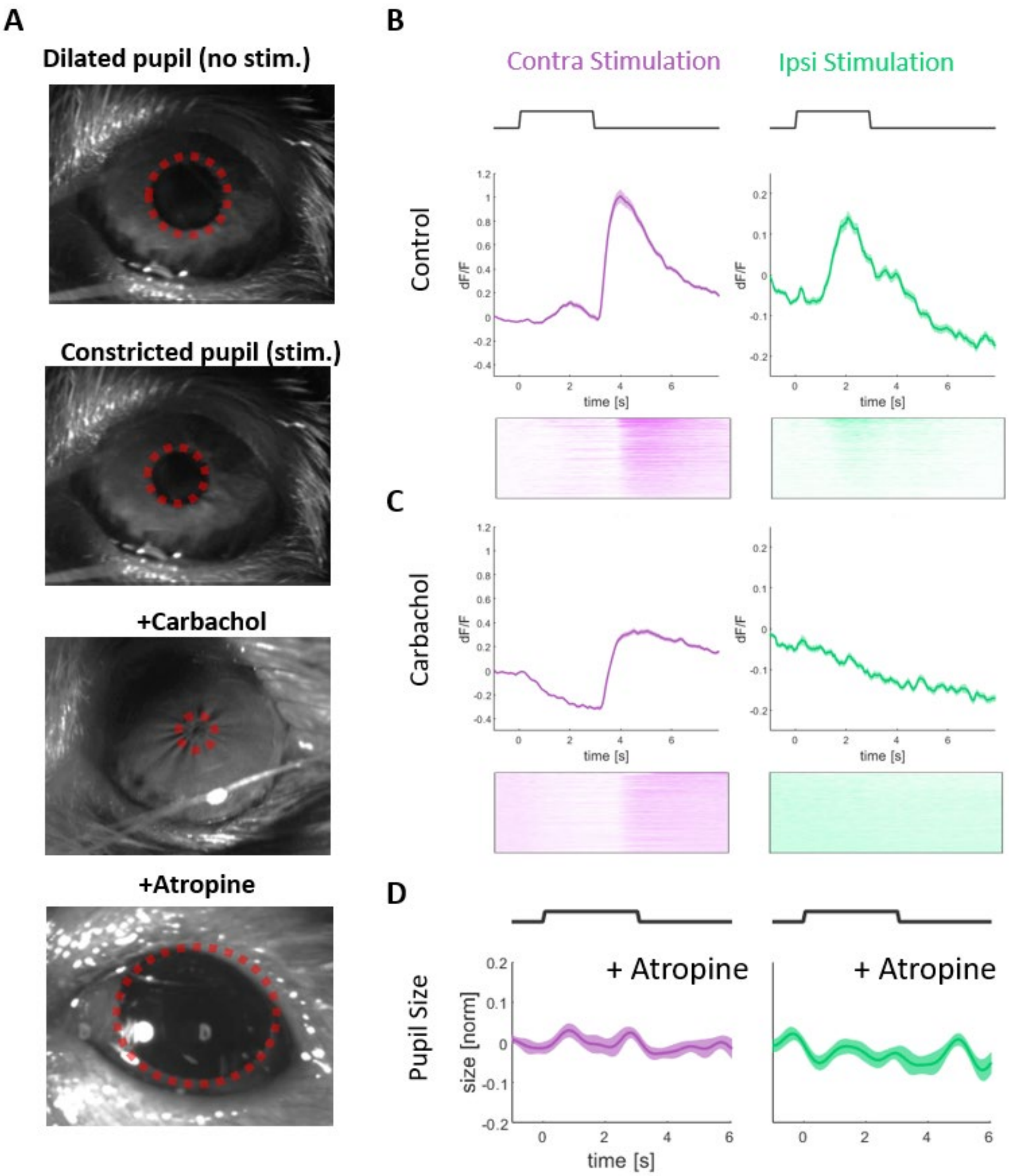

Figure S5: Pharmacological dilation of the Pupil with Atropine, contraction with carbachol. A) Example of animal pupil when dilated (top), constricted due to visual stimulation (2nd figure), constricted due to carbachol administration (3rd figure), and dilated due to atropine administration (bottom). Dotted circle indicates approximate extent of pupil opening. B) Population activity (top) and heatmap (bottom) of OFF RGC activity before Carbachol administration. C) Same boutons in B after carbachol administration. D) Pupil size after Atropine administration, showing no pupil change to either eye stimulation due to full-field flash. N = 4 animals.

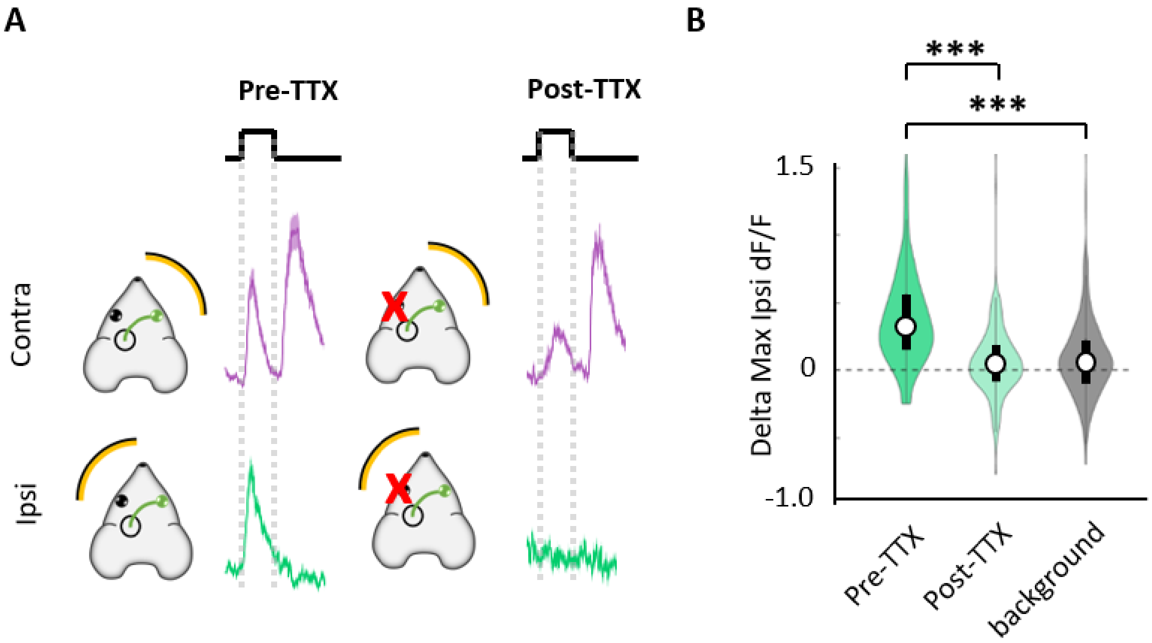

Figure S6: Inactivation of ipsilateral eye with TTX abolishes pupil-mediated binocular RGC responses but not monocular RGC responses.
A) Left: Population OFF response to contralateral stimulation (top, violet) and ipsilateral stimulation (bottom, green) (n=187 boutons, N=3 animals). Note responses to ipsilateral stimulation disappear after inactivation of ipsilateral eye (bottom right). Right: Same as left, but after TTX inactivation of ipsilateral eye (n=171 boutons, N=3 animals). B) Difference between maximal response before and during onset of population before and after TTX inactivation of ipsilateral eye. ***p <0.001

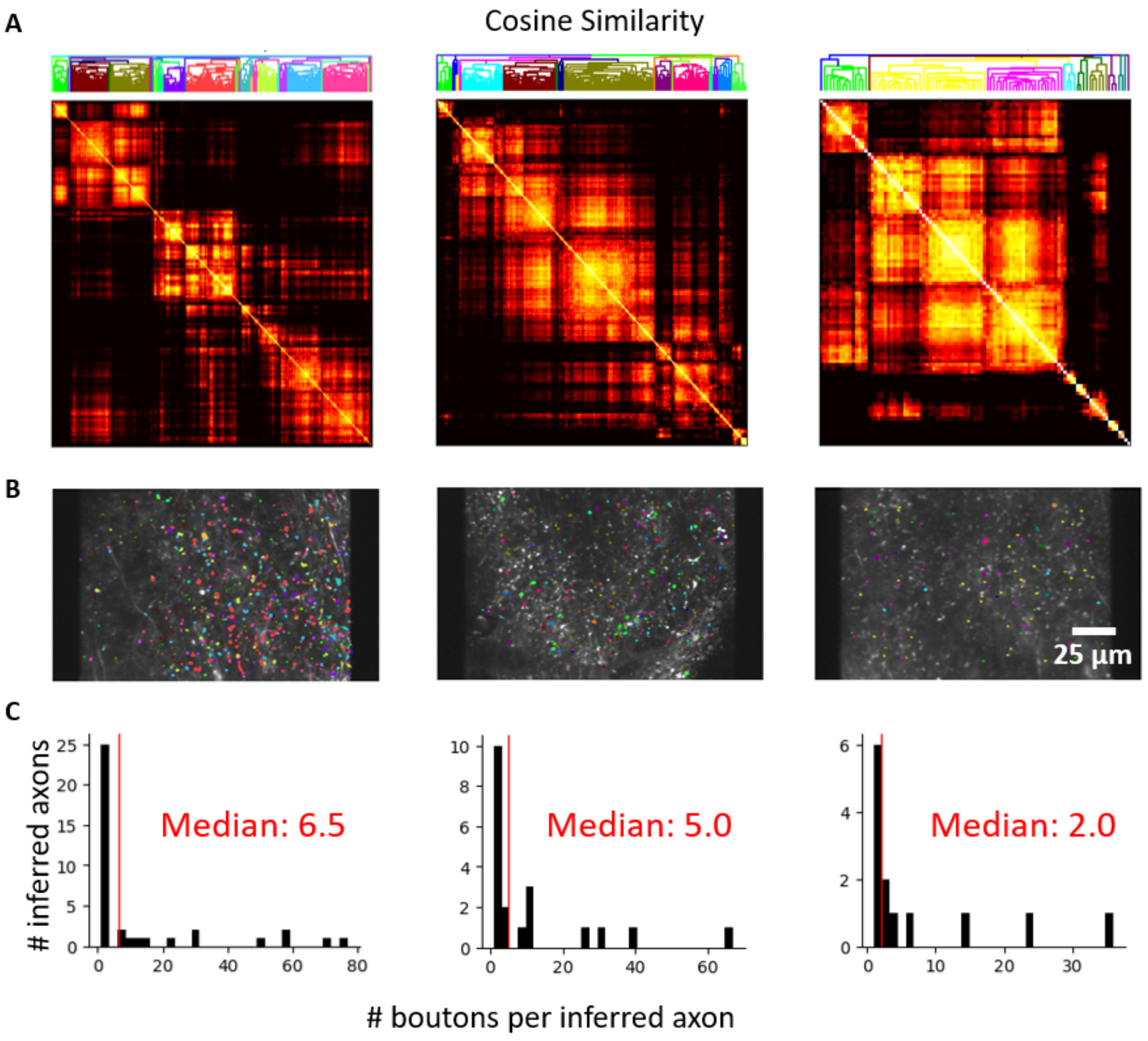

Figure S7: Axon identification analysis performed over 3 different field of views.
A) Bottom: pairwise cosine similarity (1-cosine distance) matrices calculated between the spontaneous activity correlation vectors, which reflects how much different boutons are similar in their correlation with all the other boutons. Top: dendrogram representing the clusters of boutons obtained from the WPGMA linkage clustering using the cosine distance matrix. B) The 3 field of views used for the analysis. Boutons' masks are color-coded according to the cluster identity. C) Distributions of the cluster sizes, i.e number of boutons per inferred axon. The red lines are on the median of each distribution.

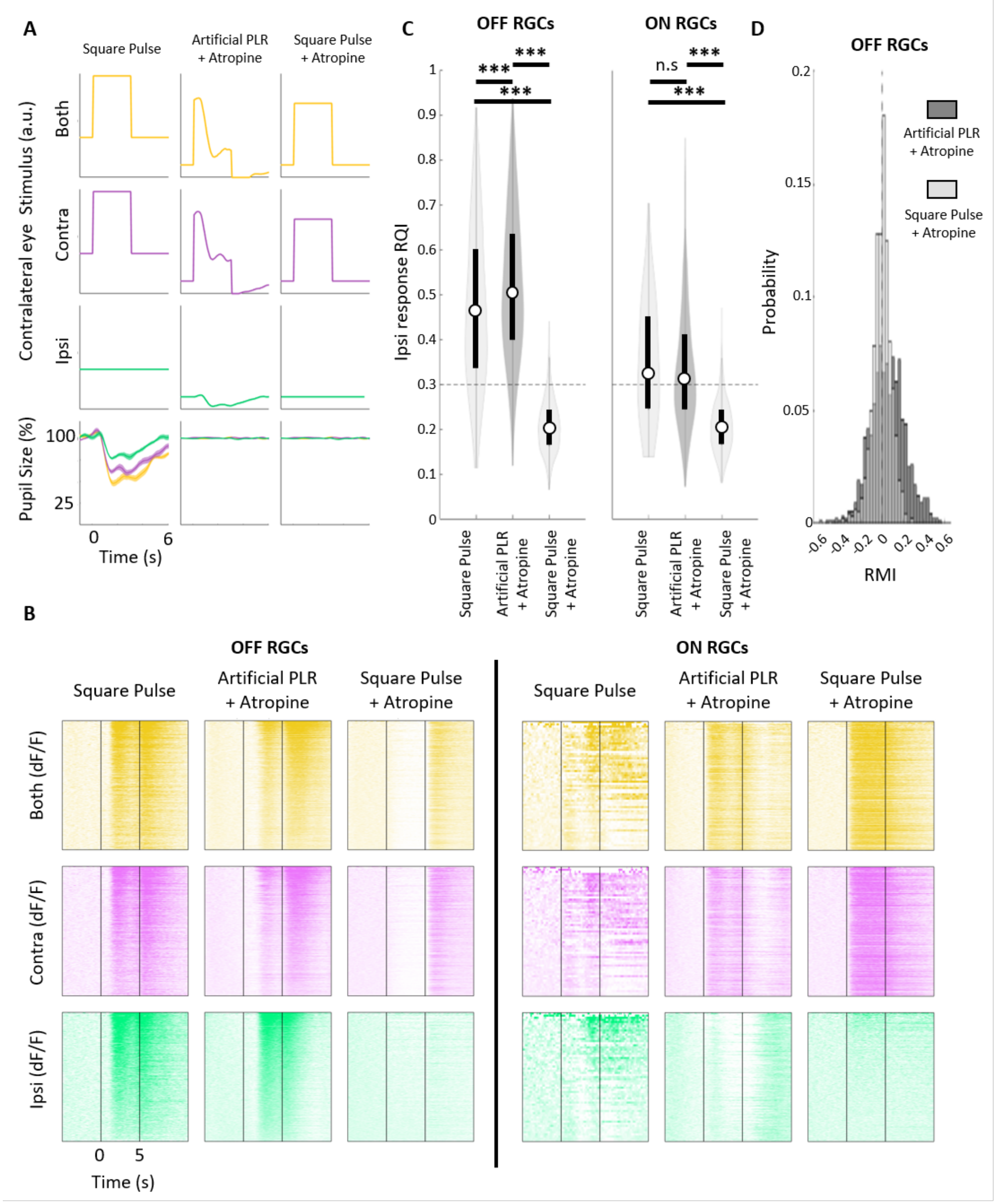

Figure S8: Luminance dimming modeling pupil constriction. A) Stimulus design. Left: Control experiment. Presenting contralateral, ipsilateral, or binocular stimulation. Middle: The application of atropine to the contralateral eye prevented PLR. Animals were presented with binocular, contralateral, and ipsilateral stimulation, modeling luminance change during PLR. Right: The application of atropine to the contralateral eye prevented PLR. Stimulation corrected only for

increased pupil size. B) OFF RGC bouton (leftmost 3 columns, N=3 animals, n=899 boutons) and ON RGC boutons (rightmost 3 columns, N = 2 animals, n = 61 boutons) responses to binocular, contralateral and ipsilateral stimulation (N=2 animals, n=61, 517, and 780 boutons per respective column). Left: square pulse without atropine. Middle: After atropine application, responses to pupil-modeled stimulation (N=3 boutons, n=887 animals), Right: After atropine application and square pulse presentation. Heatmap scale -0.3 dF/F to 2 dF/F. C) Response quality indices (RQI) of responses to ipsilateral stimulation presented in B. ON RGCs control to luminance modeled p-value = 0.51. All other p-values <0.001, ANOVA. D) Response modulation index (RMI) for Atropine + modulated stimulus (mean= 0.055, p<0.001) and Atropine (mean=-0.004, p=0.22, t-test).

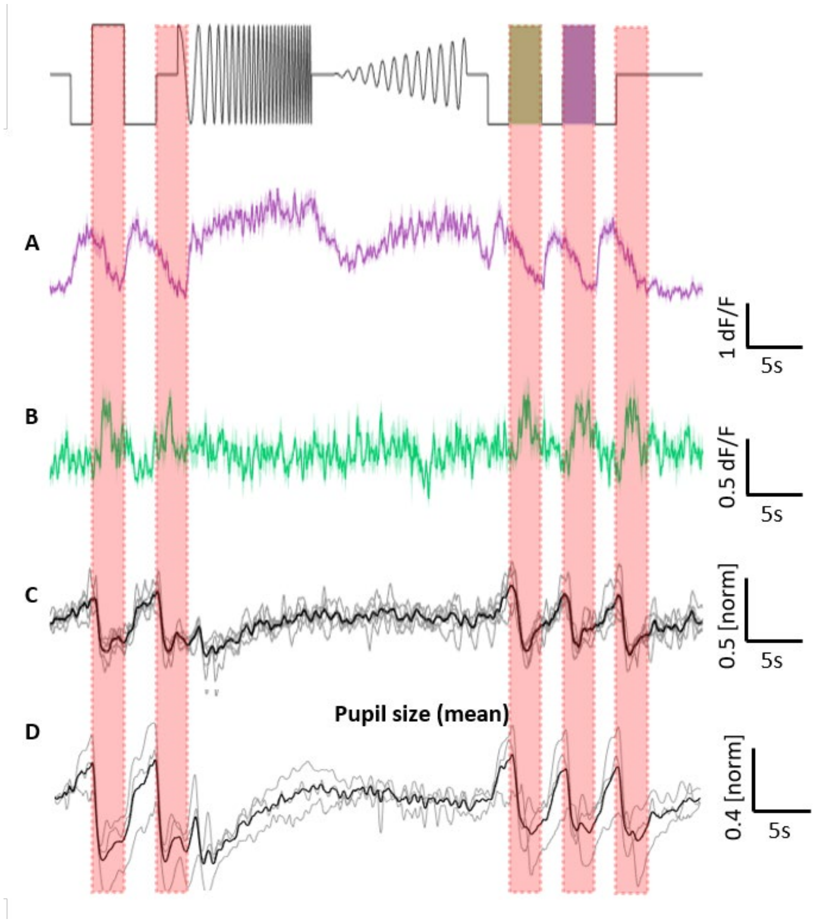

Figure S9 : PLR during chirp stimulation A) Response of example RGC to contralaterally-presented chirp stimulus. B) Response of same bouton as in A to an ipsilaterally-presented chirp stimulus. C) Pupil dynamics of animal's contralateral pupil during recording of RGC axon A-B. D) Mean of 3 animals' pupil activity when presented with the chirp stimulus. Note lack of pupil activity in C and D at the frequency and amplitude ramp component of the chirp stimulus, the chirp amplitude ramp being at 2Hz and thus sup-optimal for pupil stimulation (see Fig. 6). Red rectangles are increases in luminance that induce a PLR.

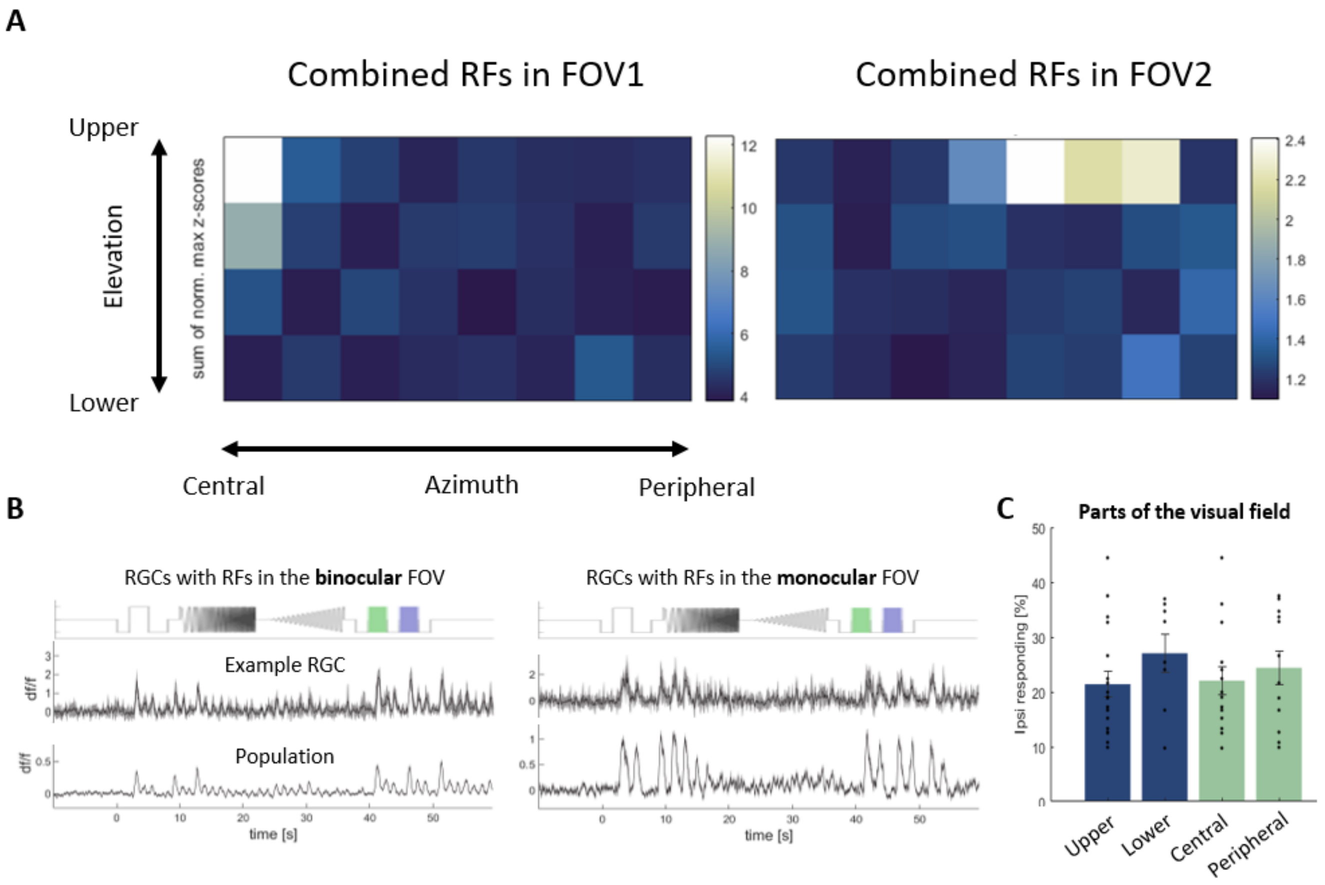

Figure S10: Pupil-induced binocular responses occur in all receptive fields
A) Left: Summary of receptive fields in example FOV with boutons viewing the front of the visual scene. For each stimulus location on 4x8 grid heatmap shows sum of normalized maximal z-scores of visual responses of all RGCs. Right: Same as left for another example FOV. FOV 1 n=210 boutons, FOV 2 n=55 boutons) B) Example RGC and Population response of all OFF boutons in FOVs shown in A to ipsilateral chirp stimulation. C) Percentage of boutons exhibiting PLR-driven responses in FOVs with different receptive fields. n= 5028 boutons, N=11 animals.

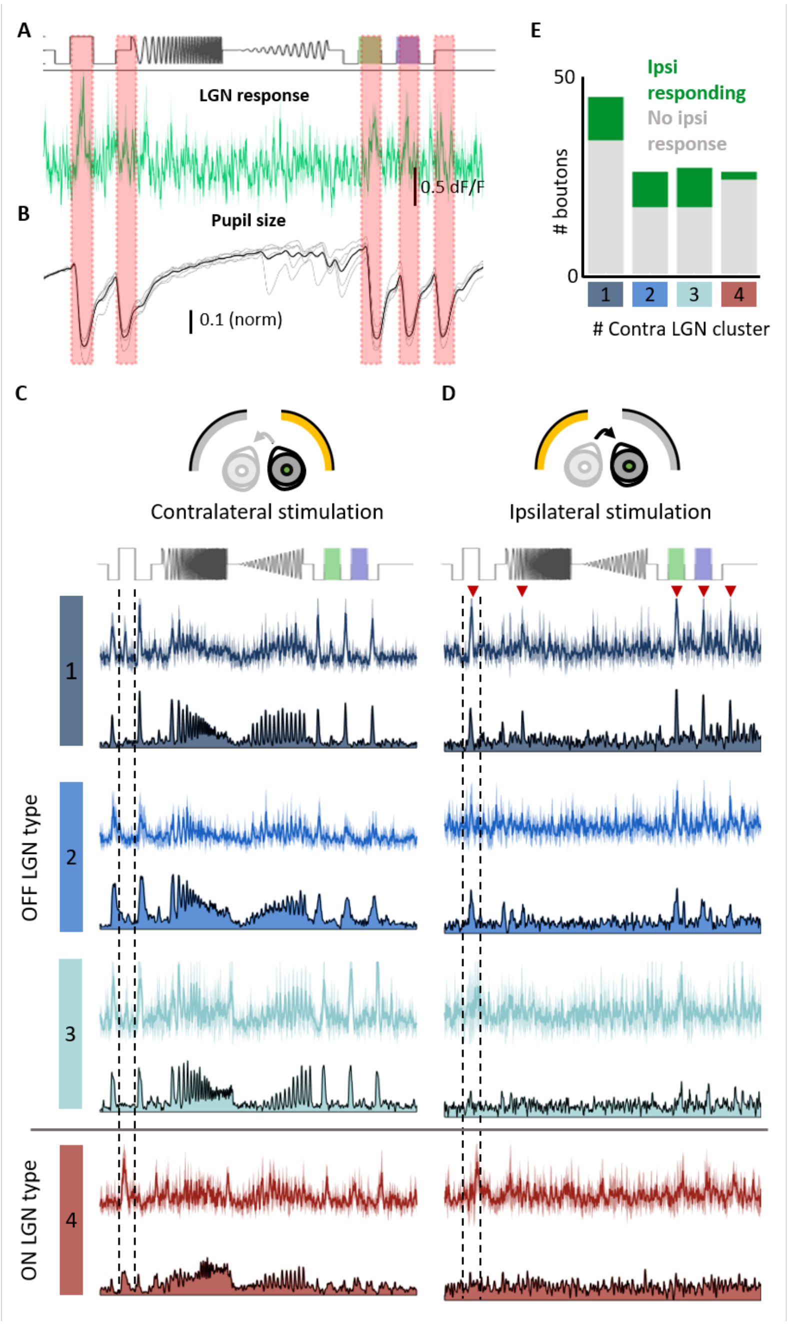

Figure S11: LGN bouton responses to chirp stimulation. A) Example LGN bouton responding to ipsilateral stimulation (mean +/- SEM). B) Pupil constriction in response to chirp stimulation. Dark gray: mean response. Light gray: each repetition. Red square: instances of sharp increases in light in chirp that can drive a PLR. C) Responses of dLGN axons to chirp stimulus presented to the contralateral eye, producing 3 OFF (n= 43, 26, 28 boutons respectively) and 1 ON class (n=27). Top row is example bouton, bottom is population average. D) Responses of each dLGN cluster in C to chirp presentation to the ipsilateral eye. Arrowheads = responses consistent with pupil-induced responses. Note delay from stimulus onset (dashed lines). E) Prevalence of ipsilateral responses in the 4 contralaterally-determined dLGN axons classes.

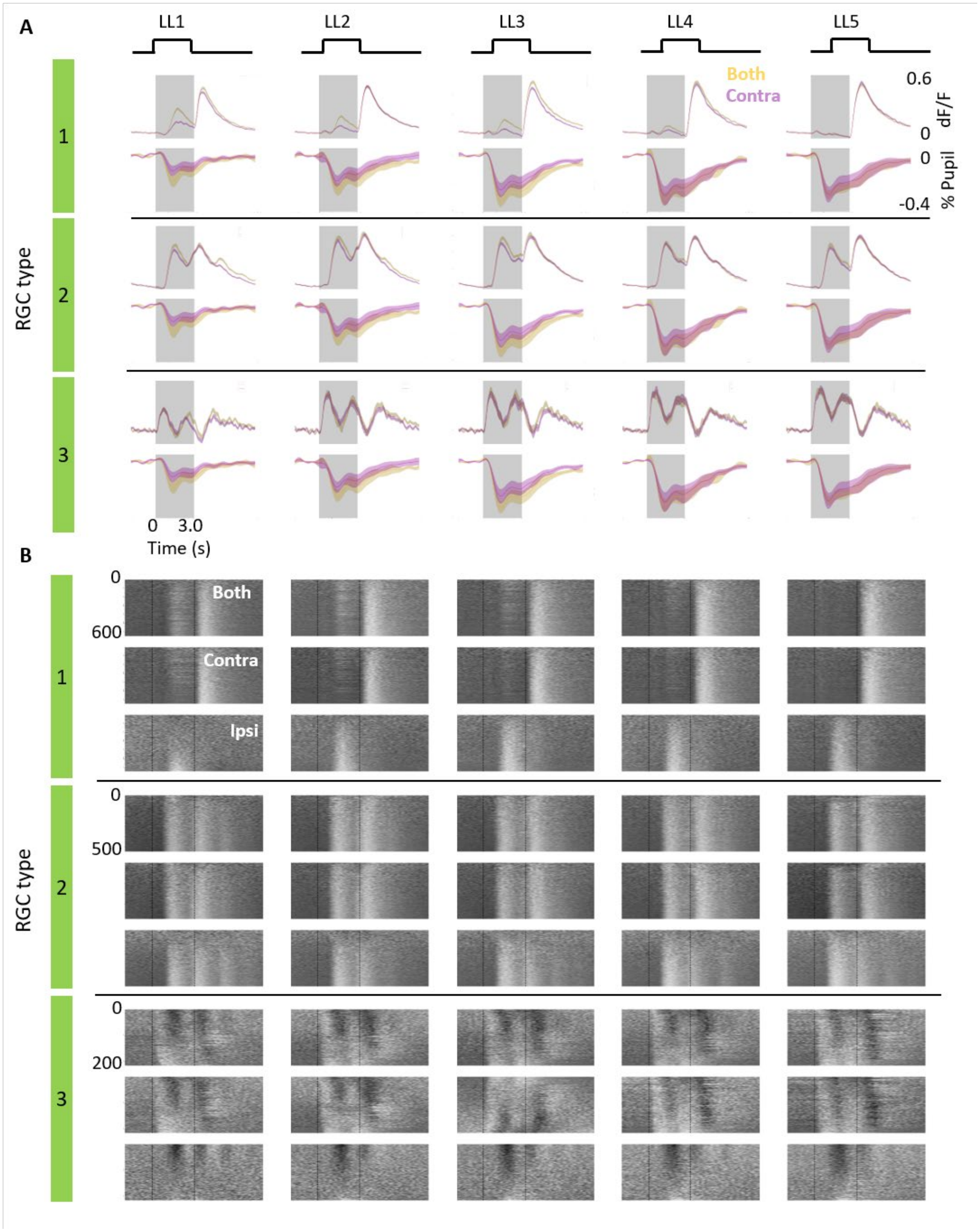
A
LL1
LL2
LL3
LL4
LL5
Both
Contra
0.6
0
dF/F
0
-0.4
% Pupil
RGC type
1
2
3
0
3.0
Time (s)
B
0
600
Both
Contra
Ipsi
500
200

Figure S12: RGC population responses to different luminance steps presented contralaterally or binocularly. A) Trial average responses to 5 different luminance steps LL1-LL5, namely 12, 20, 41, 44, 65 LUX, stepping from a baseline luminance of 9.5 LUX. Each horizontal section identified by the colored rectangles on the left report the responses for a specific functional population: 1-2 are OFF-type RGC populations, while 3 is a ON-type RGC population. For each section, the traces on top report the population averages of the responses for the 5 luminance steps, together with the standard error (shaded area); the traces on the bottom of each section report the trial-averaged % change in the pupil size during the presentation of each stimulus. The yellow color denotes responses recorded when the stimuli were presented to both eyes (binocular stimulation), while the purple traces denote the responses recorded when the stimuli were presented only to the contralateral eye. B) Heatmaps reporting the trial-averaged responses for all the boutons belonging to each of the 3 functional populations. The layout logic is the same as in A); for each population, responses to binocularly, contralaterally and ipsilaterally – presented stimuli are displayed, from top to bottom.

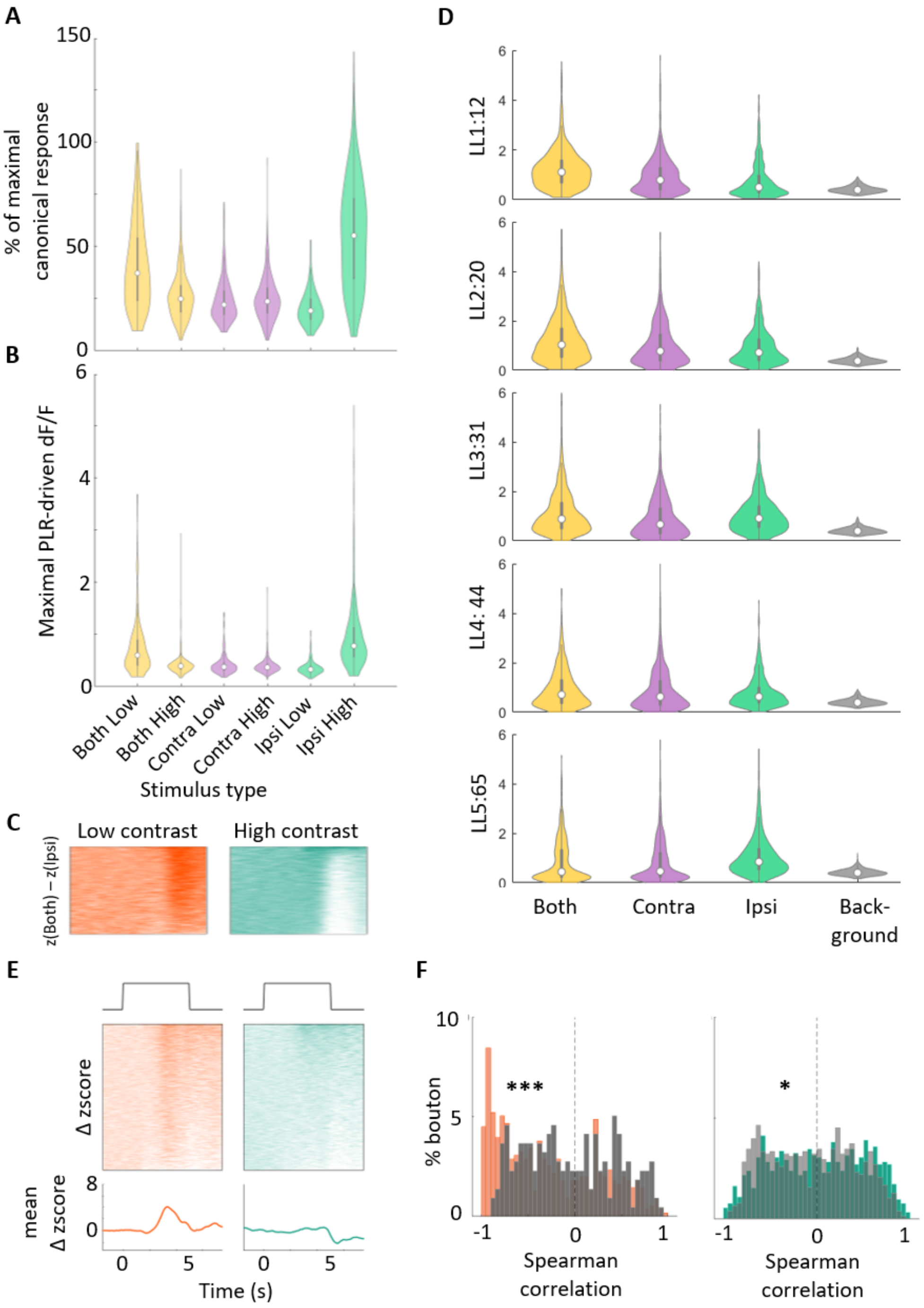

Figure S13: Contrast-dependent binocular facilitation in RGC boutons. A) Response strength of PLR-driven responses in a subpopulation of OFF RGC boutons (shown in detail in Figure 5). dF/F of PLR-driven response as a percentage of canonical response during low and high contrast binocular (yellow), contralateral (purple), and ipsilateral (green) stimulation. B) Same as A, but showing maximal dF/F of PLR driven responses. C) Frame-by-frame difference in z-score between response to binocular stimulation and response to ipsilateral stimulation during low-contrast (orange) and high-contrast (green) flash stimulation in the OFF subpopulation further described in

figure 5. D) Maximal dF/F of PLR-driven response of all contralateral OFF RGC boutons to all luminance steps (LL1: 12 LUX n=714 boutons ; LL2: 20 LUX, n=861 boutons; LL3: 31 LUX, n= 911 boutons; LL4: 44 LUX, n=909 boutons and LL5: 65 LUX, n=920 boutons) during binocular stimulation (yellow), contralateral stimulation (purple) and ipsilateral stimulation (green). Gray violin plot represents negative control, maximal response before stimulation. E) Top: Frame by Frame difference between z-score of response to binocular stimulation and contralateral stimulation during low contrast (20 LUX, orange) and high contrast (65 LUX, green) stimulation for all OFF boutons. Bottom: Mean of z-score difference shown on top. F) Spearman correlation between frame-by-frame binocular facilitation of all OFF boutons (shown in E) and frame-by-frame difference in strength of PLR in corresponding pupil during binocular and monocular stimulation. Left: Low contrast stimulation before (orange) and after atropine (gray), two-sample t-test: $p<0.001$. Right: Same as left, but high contrast stimulation, two-sample t-test: $p=0.026$.

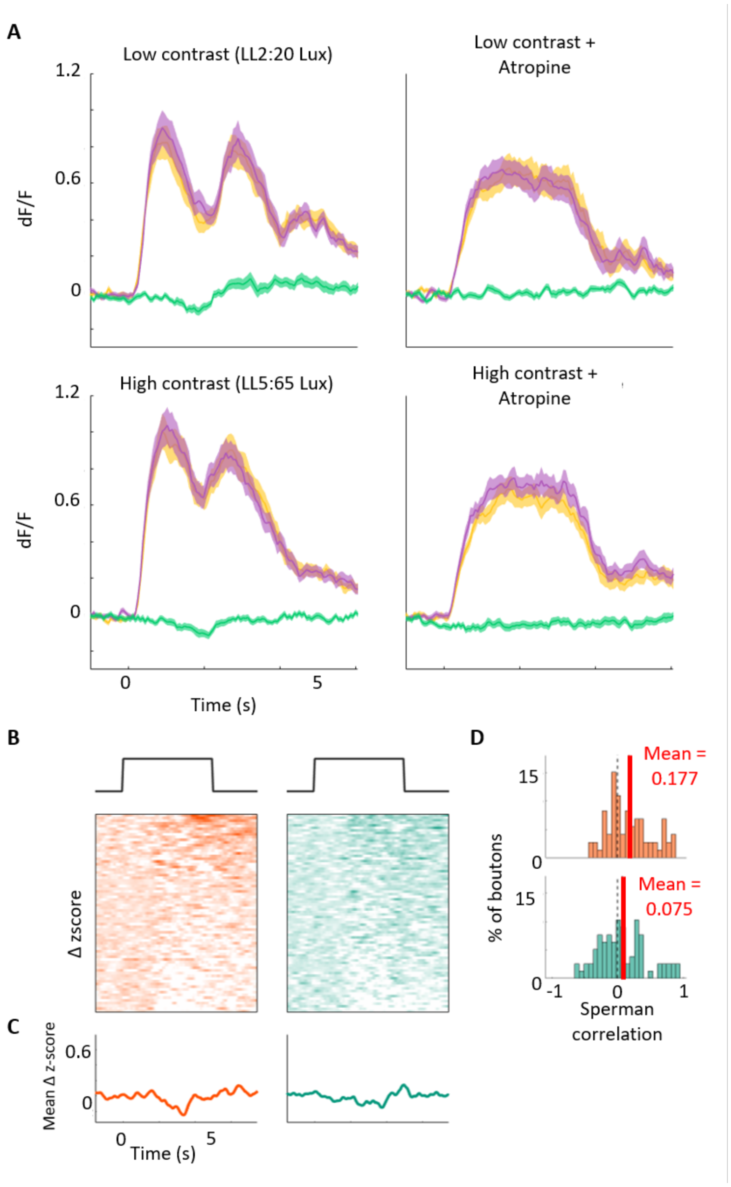

Figure S14: ON RGC boutons show no binocular modulation. A) Top Left: Population response of contralateral ON RGC boutons to binocular (yellow), contralateral (violet) and ipsilateral (green) low contrast stimulation (N=5 animals, n=73 boutons). Top Right: Same as top right after application of atropine (N=5 animals, n=73 boutons). Bottom Left: Same as Top Left during high contrast stimulation (N=5 animals, n=78 boutons). Bottom Right: Same as Bottom Left after application of atropine (N=5 animals, n=72 boutons). B) Frame by Frame difference between z-score of ON RGC responses to binocular stimulation and contralateral stimulation during low contrast (20 LUX, orange) and high contrast (65 LUX, green) stimulation. C) Mean of z-score difference shown in B. D) Spearman correlation between contralateral z-score of visual response of ON boutons and z-score of pupil size between 0.5s after the onset of the stimulation to the end of the stimulation. Top: Low contrast stimulation (mean= 0.177, one sample t-test, $p<0.001$). Bottom: High contrast stimulation (mean=0.075, one-sample t-test, $p=0.08$).

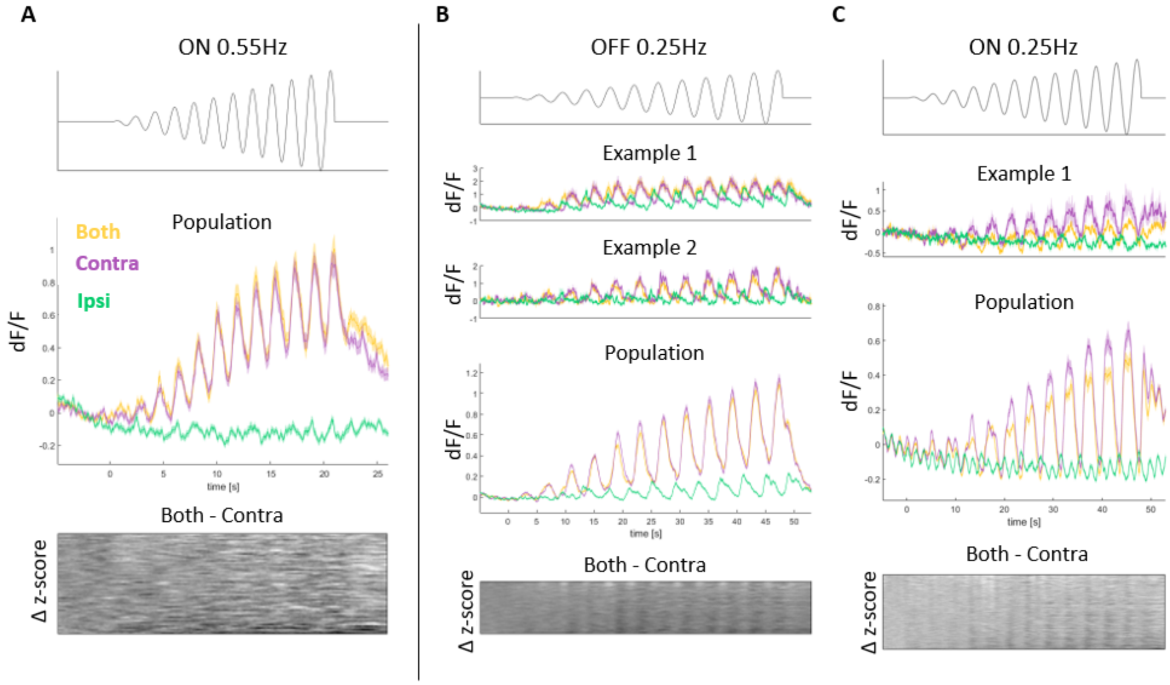

Figure S15: Responses to 0.55Hz and 0.25 Hz sinusoidal contrast ramp. A) Top: Population response of ON RGC boutons (N=2 animals, n=57 boutons) to 0.55Hz sinusoidal contrast ramp presented to both eyes (yellow), contralateral eye (violet), or ipsilateral eye (green). Bottom: Frame-by-frame difference in z-score between response to binocular and contralateral stimulation for all boutons B) Responses of OFF RGC boutons. Top: Two example bouton responses to contrast ramp presented to both eyes (yellow), contralateral eye (purple), or ipsilateral eye (green). Middle: Population response of 316 OFF RGC boutons from 5 animals (mean +/- SEM). Bottom: Frame-by-frame difference in z-score between response to binocular and contralateral stimulation for all boutons. C) Same but for ON boutons (n=145 boutons, N=5 animals).

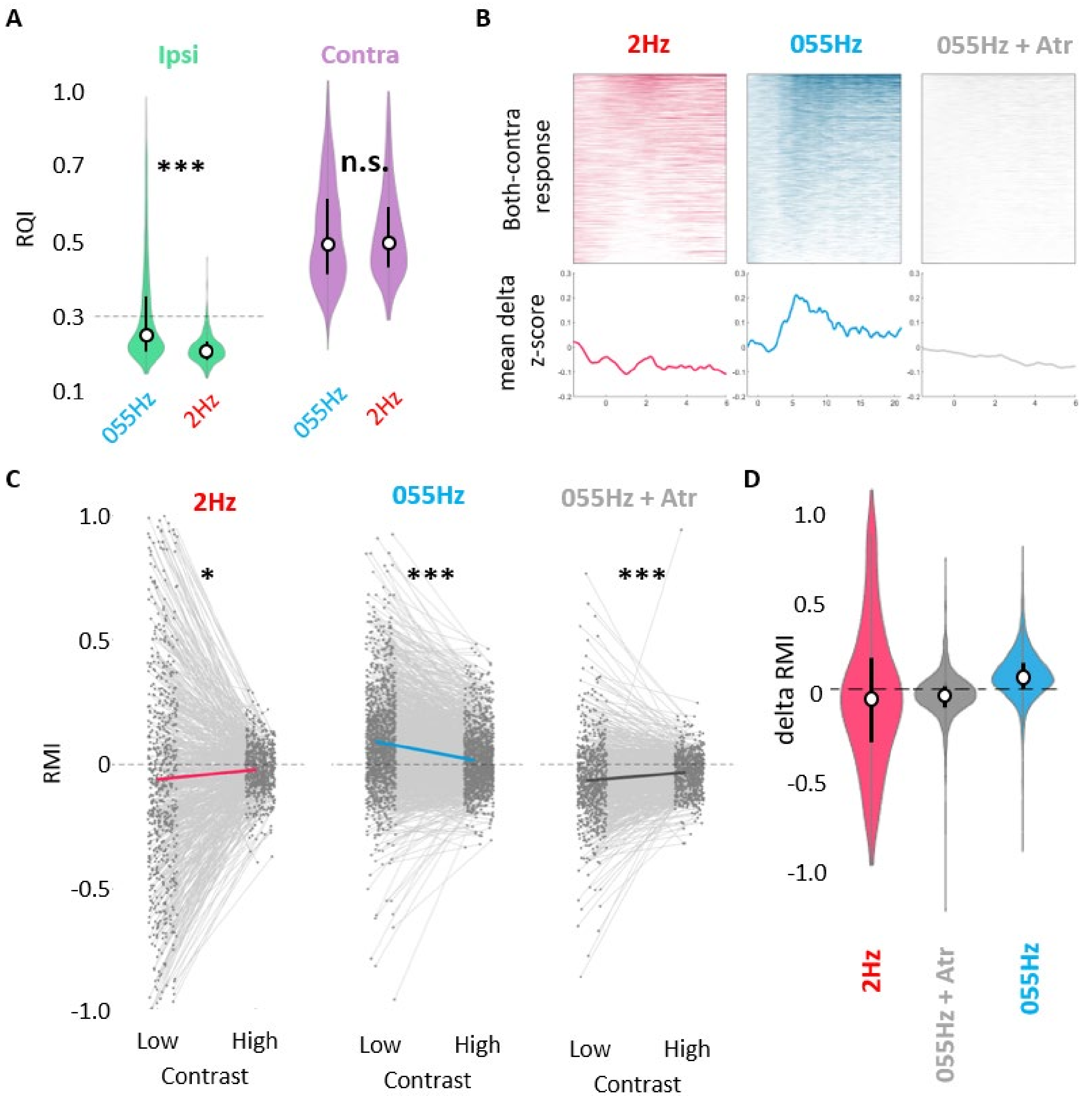

Figure S16: Frequency dependent low contrast binocular facilitation. A) Response quality index of ipsi (RQI) responses (green) to either 0.55Hz stimulation or 2Hz stimulation ($p<0.001$) and contra responses to either 0.55Hz stimulation or 2Hz stimulation ($p=0.90$). Threhold for response is a RQI of 0.3 or higher (see Methods) B) Difference between response to contralateral and binocular stimulation (as z-score) over the course of contrast ramp. Top: Heatmap of all boutons. Bottom: Mean difference over responses. Pink: 2 Hz stimulation. Blue: 0.55Hz stimulation. Gray: 0.55Hz stimulation after Atropine. C) RMI of all OFF boutons during low contrast conditions (peaks 2-5) and high contrast conditions (peaks 7-11). Line connects the two means. Left: 2 Hz stimulation ($p=0.014$) Middle: 0.55Hz stimulation ($p<0.001$). Right: 0.55 Hz + Atropine ($p<0.001$). D) Mean ±SEM of difference between high and low contrast RMI shown in C.

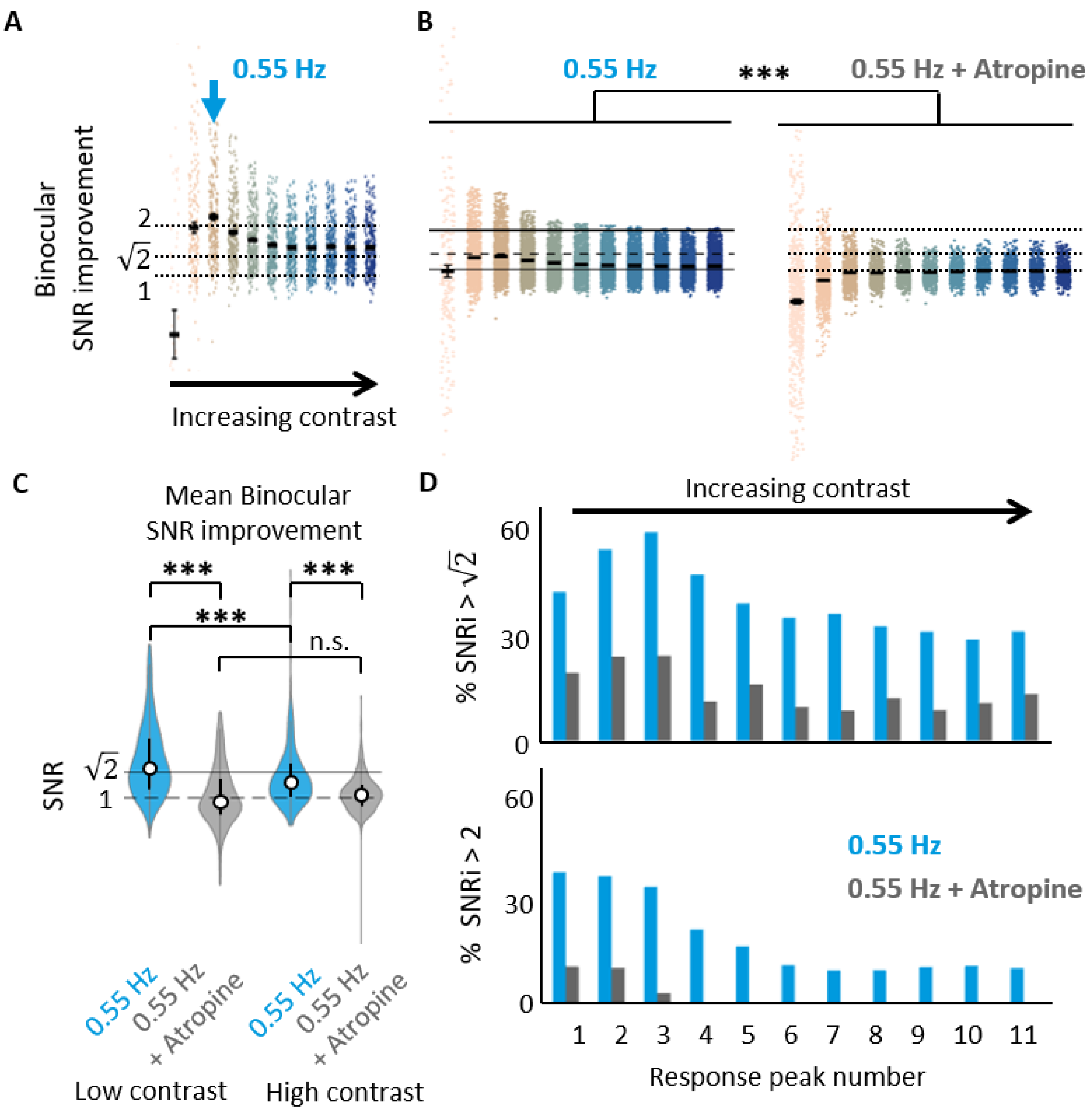

Figure S17. Pupil-induced binocular facilitation may underlie binocular summation. (A) Binocular SNR improvement in 25% most modulated RGC axons to each peak of a 0.55Hz amplitude ramp of increasing contrast. Note mean SNR improvement over a factor of 2 for the third peak (arrow). (B) RGC axon binocular SNR improvement in all RGC axons before (left) and after atropine administration (right). ***p <0.001 (t-test, Bonferroni corrected for multiple comparisons) (C) Mean Binocular SNR improvement to 0.55Hz stimulus before and after atropine in low contrast (peaks 2,3,4) and high contrast (peaks 9, 10, 11) in 50% most modulated boutons. ***p <0.001 (1-way ANOVA) (D) Percentage of all RGC boutons that exhibit binocular SNR improvement over $\sqrt{2}$ (top) or over 2 (bottom).

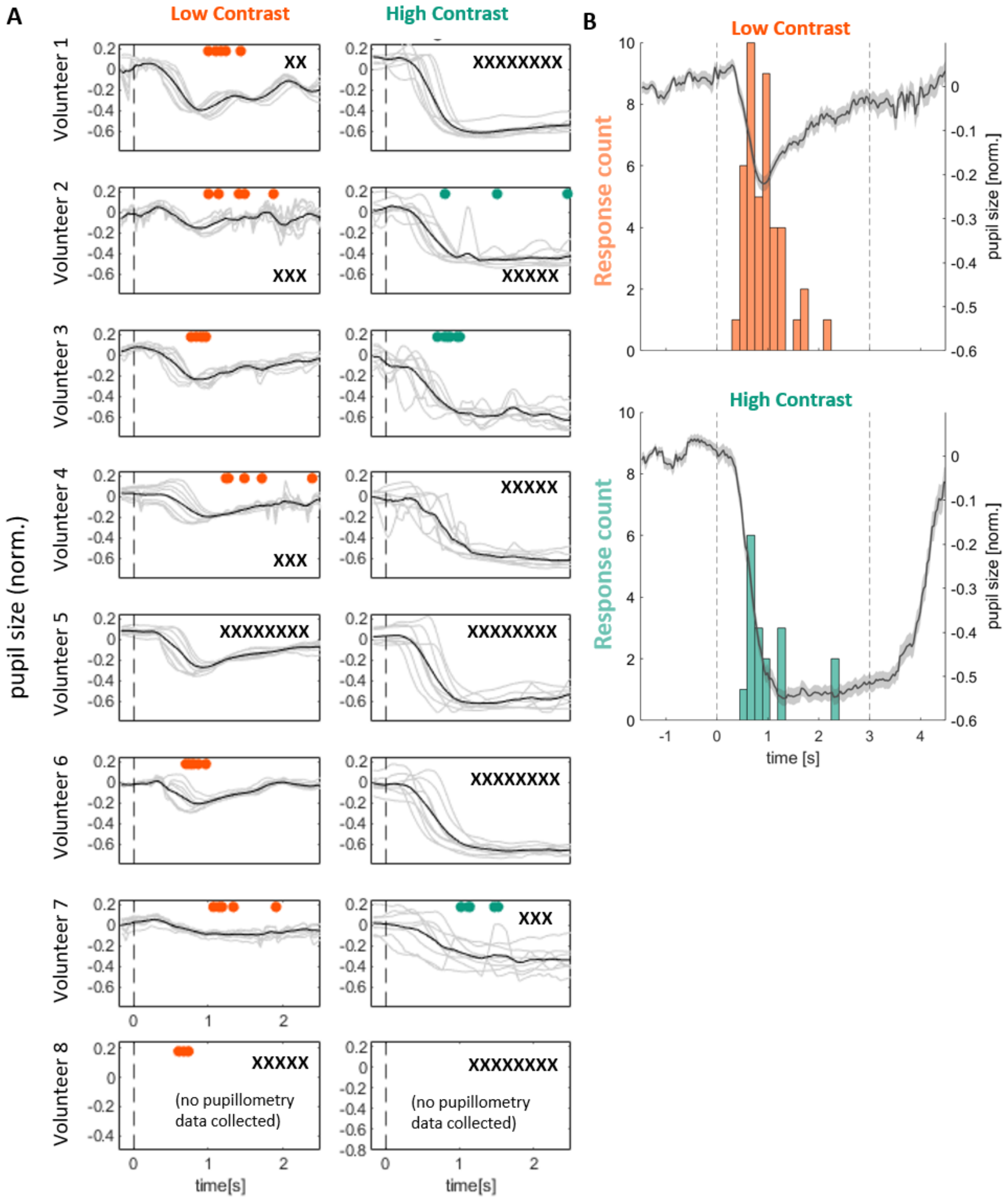

Figure S18: Human detection of PLR-induced luminance change. A) Normalized pupil size (black=mean, gray=individual trial) during the presentation of low contrast (left) or high contrast (right)full-field flash. The dotted line represents the start of the flash. Orange or green dots represent the times when luminance dimming was detected, while 'x' signs represent no detection (in total, 8 trials per contrast level). B) PLR (mean +/- SEM pupil size) and dimming detection times corrected for response time (estimated for the human at 200 ms). Low contrast flashes are from a 9.5 Lux baseline to 20 Lux, high contrast from 9.5 Lux to 65 Lux.
