# Supplementary material for: Pupil size modulation drives retinal activity in mice and shapes human perception
Source: Nat Commun. 2025 Aug 8;16:7334. doi: 10.1038/s41467-025-62736-4 (PMC12334691; doi:10.1038/s41467-025-62736-4)
Supplement: Supplementary file 2 — Reporting Summary [file 41467_2025_62736_MOESM2_ESM.pdf]

nature portfolio

Corresponding author(s): Santiago Rompani

Last updated by author(s): Nov 25, 2024

Reporting Summary

Nature Portfolio wishes to improve the reproducibility of the work that we publish. This form provides structure for consistency and transparency in reporting. For further information on Nature Portfolio policies, see our Editorial Policies and the Editorial Policy Checklist.

Statistics

For all statistical analyses, confirm that the following items are present in the figure legend, table legend, main text, or Methods section.

n/a	Confirmed	
☐	☒	The exact sample size (*n*) for each experimental group/condition, given as a discrete number and unit of measurement
☐	☒	A statement on whether measurements were taken from distinct samples or whether the same sample was measured repeatedly
☐	☒	The statistical test(s) used AND whether they are one- or two-sided<br>*Only common tests should be described solely by name; describe more complex techniques in the Methods section.*
☐	☒	A description of all covariates tested
☐	☒	A description of any assumptions or corrections, such as tests of normality and adjustment for multiple comparisons
☐	☒	A full description of the statistical parameters including central tendency (e.g. means) or other basic estimates (e.g. regression coefficient) AND variation (e.g. standard deviation) or associated estimates of uncertainty (e.g. confidence intervals)
☐	☒	For null hypothesis testing, the test statistic (e.g. *F*, *t*, *r*) with confidence intervals, effect sizes, degrees of freedom and *P* value noted<br>*Give P values as exact values whenever suitable.*
☒	☐	For Bayesian analysis, information on the choice of priors and Markov chain Monte Carlo settings
☒	☐	For hierarchical and complex designs, identification of the appropriate level for tests and full reporting of outcomes
☐	☒	Estimates of effect sizes (e.g. Cohen's *d*, Pearson's *r*), indicating how they were calculated

Our web collection on statistics for biologists contains articles on many of the points above.

Software and code

Policy information about availability of computer code

Data collection	2-photon microscopy data was obtained with custom NLW micrsocope (see methods) and with code proprietary to NLW (Scanbox)
Data analysis	Analysis was performed with suite2p for ROI detection, and custom matlab code for analysis (see link to lab github in code submission checklist for version control and additional information). link : https://github.com/tjasalapanja/PLR_RGC_analysis or https://zenodo.org/records/15709769 (DOI: 10.5281/zenodo.15709769).

For manuscripts utilizing custom algorithms or software that are central to the research but not yet described in published literature, software must be made available to editors and reviewers. We strongly encourage code deposition in a community repository (e.g. GitHub). See the Nature Portfolio guidelines for submitting code & software for further information.

Data

Policy information about availability of data

All manuscripts must include a data availability statement. This statement should provide the following information, where applicable:

- Accession codes, unique identifiers, or web links for publicly available datasets
- A description of any restrictions on data availability
- For clinical datasets or third party data, please ensure that the statement adheres to our policy

The raw data that support the mouse findings of this study are available upon request, since the two-photon recordings are several terabytes large, and thus not compatible with any existing repository. The processed data of analyzed bouton activity have been deposited in github under accession code https://zenodo.org/

records/15709769 (DOI: 10.5281/zenodo.15709769). No additional aspect of the human psychophysics data can be shared due to respective privacy policies. Source Data are provided with this paper. Any additional request for material and correspondence should be addressed to SR.

Research involving human participants, their data, or biological material

Policy information about studies with human participants or human data. See also policy information about sex, gender (identity/presentation), and sexual orientation and race, ethnicity and racism.

Reporting on sex and gender	for mice: both male and female mice were used in equal ratios throughout the study, with no sex differences found in all recorded data. For human experiment in figure 7, three male volunteers were used. For figure 8,4 male and 4 female volunteers were obtained.
Reporting on race, ethnicity, or other socially relevant groupings	We used no socially constructed or socially relevant categorization--the tested parameters are expected to be a simple visual reflex that is widely consistent between sex, gender, and any such socially relevant categorization. The analysis is between two forms of stimuli that are internally controlled and match expected mouse data where there is overwhelmingly no sex difference. For data in Figure 8, we asked their gender, with 4 reporting male, 4 female.
Population characteristics	All 11 human volunteers were scientists working in the lab or adjacent labs to one of the authors, and were of mixed ancestry and gender. Note the narrow pool of participants this was obtained from (EMBL Rome colleagues for Figure 8 and Helsinki University for figure 7), and that this could lead to confounds due to such a shared environmental setting and educational backgrounds. We also did not collect volunteer age and other covariants due to the need to ensure volunteer privacy, given they were obtained from a small pool of local volunteers.
Recruitment	local volunteers in the labs (both Helxinki University and EMBL Rome). This of course may bias the findings, since recruitment was not unbiased nor designed to be cross-sectional of the general population, but of readily available researchers willing to volunteer.
Ethics oversight	The human study in figure 7 was conducted in accordance with the principles of the Declaration of Helsinki and the guidelines of University of Helsinki,as per the Research Ethics Committee of the Faculty of Medicine.<br><br>Figure 8 approval was obtained from: EMBL BIAC (Bioethics Internal Advisory Committee) and DPO (Digital Protection Office) as per IP68 (EMBL Internal Policy, section 68) guidelines (approved study reference DPR-2023-008).

Note that full information on the approval of the study protocol must also be provided in the manuscript.

Field-specific reporting

Please select the one below that is the best fit for your research. If you are not sure, read the appropriate sections before making your selection.

☒ Life sciences ☐ Behavioural & social sciences ☐ Ecological, evolutionary & environmental sciences

For a reference copy of the document with all sections, see nature.com/documents/nr-reporting-summary-flat.pdf

Life sciences study design

All studies must disclose on these points even when the disclosure is negative.

Sample size	No sample size calculation was performed, due to the technical difficulty in obtaining efficient mouse infection, craniotomies, and habituation to obtain robust recordings, all which could yield very variable number of recorded cells per animal or field of view. Post hoc analysis was performed to determine if statistical significance was obtained, which it overwhelmingly was, with all the key findings in our study having statistical significance of less than 0.001. This is common in circuit neuroscience, where robust findings can be obtained from a small number of animals, but obtaining high-quality animals is technically challenging due to difficulties listed above, making a priori sample size calculation not practically viable. For human psychophysics studies, we simply performed the largest number of recordings we could practically obtain with local volunteers, expecting the effect to be very robust even with minimal numbers, and indeed it is what we observed for both data in figures 7 and 8.
Data exclusions	No data was excluded for the humans. Mice were excluded from the study if they fell sick during recording period or failed to elicit light responses due to technical issues (e.g. virus failed to express the reporter at high enough levels due to a faulty virus preparation batch).
Replication	Mouse experiments were replicated in multiple batches of experiments, both in terms of using multiple animals per finding (between 3 and 12, with usually two fields of view per animal), and with each reported response being an average of several individual responses (minimum 3, typically 5 or 6). Human data in figure 7 were confirmed in 2 separate experiments, only one of them shown in the final figure. Figure 8 data was replicated 8 times for each participant, as shown in the figure and discussed in the text. All attempts at replication were successful for both mouse and human data.
Randomization	For mice, allocation was random, as is routine for the field and does not need to be specified, since mice are inbred lines. For humans, there was no allocation, since experiment was internally controlled (all volunteers got the same stimulus set and we compared between low and high contrast responses within a volunteer, meaning we did not have control and experimental groups), as was described in the methods section.

Blinding	Not relevant for mouse data, since data was machine-quantified in identical ways using a simple readout of pupil size and comparing fluorescent responses (see methods), removing any qualitative human assessment from data analysis that would require blinding.<br>For human psychophysics data, No group allocation was performed—the data was internally controlled, comparing each participant's low contrast and high contrast responses. Blinding analysis to this condition would not work, since kinetics of pupillary response would make it immediately apparent if stimulus is low or high contrast, breaking the blind for the experimenter conducting this analysis.

Reporting for specific materials, systems and methods

We require information from authors about some types of materials, experimental systems and methods used in many studies. Here, indicate whether each material, system or method listed is relevant to your study. If you are not sure if a list item applies to your research, read the appropriate section before selecting a response.

Materials & experimental systems

n/a	Involved in the study	
☐	☒	Antibodies
☒	☐	Eukaryotic cell lines
☒	☐	Palaeontology and archaeology
☐	☒	Animals and other organisms
☒	☐	Clinical data
☒	☐	Dual use research of concern
☒	☐	Plants

Methods

n/a	Involved in the study	
☒	☐	ChIP-seq
☒	☐	Flow cytometry
☒	☐	MRI-based neuroimaging

Antibodies

Antibodies used	chicken-anti- GFP primary polyclonal antibody diluted 1:1000 (Abcam, cat. ab13970). Goat anti-chicken Alexa Fluor488, Abcam, cat. num. ab150169) diluted 1:500. DAPI (Thermo Fisher Scientific, cat. D1306, diluted 1:1000)
Validation	Previous extensive use in reference 48 of paper: Rompani, S.B., Müllner, F.E., Wanner, A., Zhang, C., Roth, C.N., Yonehara, K., and Roska, B. (2017). Different Modes of Visual Integration in the Lateral Geniculate Nucleus Revealed by Single-Cell-Initiated Transsynaptic Tracing. Neuron 93, 767-776.e6. https://doi.org/10.1016/j.neuron.2017.01.028.

Animals and other research organisms

Policy information about studies involving animals; ARRIVE guidelines recommended for reporting animal research, and Sex and Gender in Research

Laboratory animals	mice (C57BL/6-J background). Animals were housed using a 12:12-hour light/dark cycle, with lights on from 7:00 AM to 7:00 PM, housed at temperatures between 20–22?°C, and with relative humidity ranging from 40% to 60%. Source of C57BL/6 animals are internal breeding colonies. The breeders are purchased from Charles River and colony is refreshed every 2 years to avoid a genetic drift.
Wild animals	N/A
Reporting on sex	both sexes were used in mouse experiments, no sex differences found
Field-collected samples	N/A
Ethics oversight	All the mouse experiments were carried out in accordance with European Union Directive 2010/63/EU and under the approval of the EMBL Animal Use Committee and the Italian Ministry of Health License 23- 004_RM_SR. The human study was conducted in accordance with the principles of the Declaration of Helsinki and the guidelines of the University of Helsinki ethical review board. See methods for more information.

Note that full information on the approval of the study protocol must also be provided in the manuscript.

Plants

Seed stocks	N/A
Novel plant genotypes	N/A
Authentication	N/A
